# The neuroprotective effects of Liuwei Dihuang medicine in the APP/PS1 mouse model are dependent on the PI3K/Akt signaling pathway

**DOI:** 10.3389/fphar.2023.1188893

**Published:** 2023-10-18

**Authors:** Ye Yuan, Yamei Liu, Li Hao, Jinlian Ma, Simai Shao, Ziyang Yu, Ming Shi, Zhenqiang Zhang, Zijuan Zhang

**Affiliations:** ^1^ Academy of Chinese Medical Sciences, Henan University of Chinese Medicine, Zhengzhou, Henan, China; ^2^ School of Basic Medical Sciences, Henan University of Chinese Medicine, Zhengzhou, Henan, China

**Keywords:** Alzheimer’s disease, APP/PS1 mouse, Liuwei Dihuang medicine, PI3K/AKT, Aβ

## Abstract

Alzheimer’s disease (AD) is an age-related neurodegenerative disease that progressively impairs cognitive function and memory. The occurrence and development of Alzheimer’s disease involves many processes. In response to the complex pathogenesis of AD, the Traditional Chinese medicine formula Liuwei Dihuang Pill (LWD) has been shown to improve the cognitive function of AD animal models. However, the active ingredients and mechanism of action of LWD have not been fully elucidated. In this study, network pharmacological analysis predicted 40 candidate compounds in LWD, acting on 227 potential targets, of which 185 were associated with AD. Through network pharmacological analysis, the mechanism of action of LWD therapy AD is related to the inhibition of inflammatory response, regulation of neuronal state, and autophagy. In this experiment, LWD was detected in the APP/PS1 transgenic mouse model. The objective was to observe the effects of LWD on hippocampal learning and memory ability, Aβ clearance, autophagy and inflammatory response in APP/PS1 mice. The results showed that LWD improved long-term memory and working memory in APP/PS1 mice compared with the WT group. At the same time, LWD can increase the expression of hippocampal autophagy biomarkers, reduce the precipitation of Aβ, and the activation of microglia and astrocytes. Its mechanism may be related to the regulation of the PI3K/Akt signaling pathway. Thus, we demonstrate for the first time that LWD has a neuroprotective effect on APP/PS1 mice and provide theoretical foundation for the development of a new clinical treatment for AD.

## 1 Introduction

Alzheimer’s disease (AD) is a neurodegenerative disease with impaired spatial learning and memory ability and cognitive dysfunction as the main clinical manifestations (Torres and Cardenas, 2020). With the advent of the aging population, the incidence of AD is also increasing. It is predicted that by 2050 there will be a case of AD every 33 s, which has become a major health and economic problem (Jia et al., 2018). However, to date, only symptomatic treatments such as donepezil hydrochloride, memantine hydrochloride and galantamine have been approved by the US FDA for the treatment of Alzheimer’s disease. Although Aduhelm was approved by the FDA in 2021, the drug company provided less information about its clinical trials (Knopman et al., 2019; Schneider, 2019). There is no effective treatment to prevent or reverse the progression of AD.

AD is mostly classified as “dementia” and “forgetfulness” in Traditional Chinese medicine books. It is believed that the pathogenesis of this disease is based on “renal deficiency and blood stasis”. According to the theory of Traditional Chinese medicine, the kidney governs the bone which produces the marrow, and the brain is the marrow sea. When the marrow sea is empty, there will be persistent dementia and clumsy movements, which are consistent with the impaired learning and memory, action dysfunction and its major imaging changes in the clinical manifestations of modern medical AD. Therefore, the application of traditional Chinese medicine nourishing Yin and kidney method to treat the disease has been widely recognized.

The Traditional Chinese medicine Liuwei Dihuang Pills (LWD), formerly known as Dihuang Pills, or the prescription of “key to therapeutic of children’s disease”. It is widely recognised in clinical practice that the medicine treats the following symptoms such as dysplasia and replenish vital essence, tonify kidney-yin and nourish the bone marrow. As a classical traditional Chinese medicine prescription, the drug has the effects of enhancing immunity, delaying aging, anti-inflammatory, and antioxidant function (Zhou et al., 2016). LWD consists of six crude herbs including Radix rehmannia, Fructus corni, Rhizoma dioscoreae, Rhizoma alismatis, Cortex moutan, and Poria cocos in a ratio of 8 : 4 : 4 : 3 : 3 : 3. Studies have shown that LWD has therapeutic benefits for hypertension, diabetes, neurosis, neurasthenia, dementia, and Parkinson’s disease (Huang et al., 2005; Xia et al., 2014; Dai et al., 2016).

For neuronal cells, autophagy is an important mechanism for clearing pathological Aβ deposition in cells and maintaining homeostasis in neuronal cells (Reddy and Oliver, 2019). Meanwhile, neuroinflammation is considered to be part of the pathogenesis of neurodegenerative diseases, including AD. The production and aggregation of Aβ reduces the phosphorylation and activity of phosphatidylinositol-3-kinase (PI3K)/proteinkinase-B (Akt) signalling pathway, which is closely related to the development of AD and neuroinflammation (Heras-Sandoval et al., 2014). Restoring impaired autophagy in AD neuronal cells and promoting autophagy and degradation of pathological proteins in neuronal cells may be the effective means of treating AD. When the major players in neuroinflammation, astrocytes and microglia, are activated, they produce inflammatory cytokines and chemokines that maintain and enhance the inflammatory condition. Previous studies have demonstrated that LWD has the effect of promoting autophagy and inhibiting the aggregation of pathological proteins (Zhu, 2020). However, it is not clear whether these effects are related to autophagy induced by activation of the PI3K/AKt pathway.

The aim of our experiment is to observe the effects of LWD on learning and memory, neuroinflammation and autophagy levels in APP/PS1 transgenic mice, and to explore the molecular biological mechanism of these changes, in order to provide new leads for the understanding of the onset and prevention of AD.

## 2 Materials and methods

### 2.1 Animals

The animals used in this experiment are all raised in the Experimental Animal Center of Henan University of Chinese Medicine. All procedures involved in this study were approved by the Animal Ethics Committee of Henan University of Chinese Medicine (Henan University of Chinese Medicine, Zhengzhou, China). 22-week-old male APPswe/PS1dE9 (APP/PS1) transgenic mice and male transgene-negative littermates of the APP/PS1 mice of the same month age were provided by Nanjing Institute of Biomedicine, Nanjing University (batch number: N000175). In this experiment, all experimental animals were acclimatized within 1 week of purchase, and the experimental animals were housed in individual ventilation systems using a single-cage housing method. The animal rooms were maintained at a room temperature of 20–25°C with a 12 h light/dark cycle. Free diet and drinking water were provided. Experimental procedures were standardized according to the relevant principles of laboratory animal welfare.

Groups: 1) WT group: 12 APP/PS1 negative male mice were administered physiological saline 10 mL/kg daily by gavage for 60 days; 2) APP/PS1 group: 12 APP/PS1 positive male mice were administered physiological saline 10 mL/kg daily by gavage for 60 days; 3) APP/PS1+LWD group: 12 APP/PS1 positive male mice were administered LWD suspension 10 mL/kg daily by gavage for 60 days; 4) APP/PS1+DZ group: 12 APP/PS1 positive male mice were administered Donepezil suspension 10 mL/kg daily by gavage for 60 days.

### 2.2 Drug administration

All the drugs were administrated by gavage, the dosages of medicines were obtained by referring to the equivalent measuring ratio of human and animal body surface areas in the “Pharmacological Experiment Methods” edited by Professor Xu Shuyun and the basis of preliminary experiments. Liuwei Dihuang Pills (batch number 18110401) and Donepezil Hydrochloride (batch number 1701069) were purchased from Zhang Zhongjing Pharmacy. Liuwei Dihuang Pills and Donepezil Hydrochloride were dissolved in saline to prepare suspensions. Each 8 pill batch of Liuwei Dihuang Concentrated Pills was equivalent to 3 g crude drug. Once a day and with the gavage volume of 10 mL/kg, the gavage dosage and donepezil hydrochloride dose was 1.365 g/(Kg•d) and 0.758 g/(Kg•d) respectively. The WT group and the APP/PS1 group were given the same volume of normal saline (0.9% NaCl), with continuous treatment for 60 days.

### 2.3 Network pharmacology analysis

The chemical composition information of Liuwei Dihuang Pills was obtained from the TCMSP database (http://tcmspw.com/tcmsp.php). According to the similarity of the chemical fingerprint of the Chinese medicine ingredients and the known drugs, the active ingredients and target genes in the formula were collected. Oral Bioavailability (OB)≥30% and Drug Likeness (DL)≥0.18 were used as filters. The OMIM database and the GeneCards database were screened to collect known AD-related targets. EXCEL software was used to screened the common targets of Liuwei Dihuang Pill and AD, and R 3.6.1 software was used to draw the Wayne diagram. Cytoscape (version 3.9.1) was used to build a network of active ingredients and associated targets for LWD. According to the core targets obtained in the relevant target network, we used the STRING network database platform (http://string-db.org/) to construct a protein-protein interaction (PPI) network, which limited species to humans and obtained related protein interaction relationship data to further elucidate the interaction of important target genes in the treatment of AD by LWD. The KEGG pathway and GO function analysis enrichment analysis (*p <* 0.05) were performed on the obtained targets via the Metascape database (https://metascape.org/), respectively, and the roles of relevant targets in biological processes, molecular functions, cellular components and pathways were analyzed. Use R software to plot bubbles and visualize GO and KEGG analysis.

### 2.4 Morris water maze (MWM)

All mice underwent MWM behavioural experiments after completion of gavage. A pool of 1.5 m in diameter was made opaque by milk, and the water surface was 0.5 cm above the “invisible platform”. After the mice were gavaged, the water temperature was maintained at 22°C, and the mice were trained for positioning and navigation. The complete test lasted for 6 days. In the experiment, the mice were released from the same point in the four quadrants of the pond, and the position of the hidden platform remained unchanged during the experiment. Each experiment is limited to 60 s. During 1–5 days of training, these mice were trained to learn the position of the hidden platform. If the platform is not found within 60 s, the computer will stop tracking, then we remove the mouse from the water and place it on the platform for 30 s. If the mouse finds the platform within 60 s and stays on the platform for 30 s, the computer will stop tracking.

The space exploration experiment was carried out after the positioning and navigation experiment in the 6^th^ day. Remove the platform placed in the second quadrant, and select the fourth quadrant farther from the platform as the water entry point allow the mouse to search for the location of the platform. The computer automatically recorded the mouse’s swimming trajectory, the percentage of time spent in the quadrant where the platform was located, and the number of times that it has crossed the platform. During the experiment, the operator was blinded to the purpose of the experiment and the treatment method. Experimental analysis of the morris water maze used SMART 3.0 version of behavioral experimental software, which provides trajectory tracking and data analysis.

### 2.5 Y maze

All mice underwent Y maze behavioural experiments after completion of gavage. The Y maze equipment consists of a computer, a camera, and a Y-shaped maze composed of three arms of equal length with an angle of 120° and a connecting area. The three arms of the Y maze are marked as 1, 2, and 3 respectively. We place the mice in the same orientation at the intersection of the 3 maze arms, and the mice were allowed to move freely in the maze for 5 min. The system automatically records the total number of mouse movements into the arms and the order in which the arms were entered. The spontaneous alternation accuracy rate of the mouse was calculated according to the total number of arm access times and the accurate alternation times of the mouse. The calculation formula is: Spontaneous alternation accuracy rate = (accurate alternation times/total arm insertion times-2)÷100%. Among them, the mouse continuously entered 3 different arms as accurate alternate once, and the formula was substituted to calculate the spontaneous alternation accuracy rate of each group of mice. Experimental analysis of the Y maze used SMART 3.0 version of behavioral experimental software, which provides trajectory tracking and data analysis.

### 2.6 Hematoxylin-eosin staining (HE staining)

After the behavioral experiment, three mice were randomly selected from each group for the HE staining. Mice were anesthetized using isoflurane, cardiac perfusion was performed with normal saline and 4% formaldehyde, the whole brain tissue was removed after perfusion, and the material samples were placed in 4% formaldehyde solution for 24 h. Conventional paraffin embedding and sectioning; the tissue section was soaked in xylene for 10min, and then soaked for 10 min after changing xylene, 50% xylene was soaked for 5 min. Absolute ethanol soaked for 5 min, replaced with absolute ethanol soaked for 5 min, then soaked in 95%, 90%, and 70% ethanol for 5 min, separately. Washed with distilled water for 2 min, then stained with eosin for 5 min and washed with distilled water. Dehydration was carried out using 70%, 85%, 95%, and 100%, with a dehydration time of 1 min per step. Finally, neuronal morphological changes were observed under microscope.

### 2.7 Transmission electron microscopy (TEM)

After the behavioral experiment, three mice from each group were randomly selected for the TEM, they were infused with 0.9% sodium chloride and 4% glutaraldehyde after isoflurane anesthesia. The brain tissue was taken out, and the CA1 area was quickly dissected and cut into tissue blocks of about 1 cm^3^. The tissue was fixed with 2.5% glutaraldehyde for 4 h, 1% osmotic acid for 2 h, dehydrated with anhydrous ethanol and acetone gradient, and then embedded and solidified after dehydration. The ultrathin section was performed on a microtome, and the ultrathin section was stained with saturated uranyl acetate for 20 min. Then, the slices were rinsed with lead citrate and dyed for 5 min, then rinsing again and drying it. The ultrastructure and morphological differences of the slices were observed and imaged on a 60 kV transmission electron microscope.

### 2.8 Immunofluorescence

After the behavioral experiment, three mice were randomly selected from each group for the immunofluorescence, they were perfused with 0.9% NaCl and 4% paraformaldehyde under isoflurane anesthesia. Mouse brain tissue was fixed in a 4% formaldehyde solution and then dehydrated in a gradient of 20% and 30% sucrose solution. The processed brain tissue is processed into slices with a thickness of 25 microns by a frozen slicer (Leica Microsystems, Wetzlar, Germany). The tissue sections were sequentially reacted with 0.3% TritonX-100, 10% goat serum, target primary and secondary antibodies to complete immunofluorescence staining. The main antibodies are: Anti-ß-amyloid (ab201060, Abcam, 1:200); Anti-GFAP (BA0056, BOSTER, 1:100); Anti-IBA1 (PB0517, BOSTER, 1:100); Goat Anti-Rabbit IgG (BM3894, BOSTER, 1:1000); Dnk pAb to Rb IgE (ab150073, Abcam, 1:1000). Finally the images were imaged using a fluorescent microscope and analysed using ZEN 2.0 software (Olympus, Japan).

### 2.9 Western blotting

After the behavioral experiment, three mice were randomly selected from each group for Western blotting. The brain tissue of the mice was quickly removed and the hippocampus tissue was separated at low temperature. The treated brain tissue requires extraction of histones at low temperature, followed by grinding and centrifugation of the sample. The protein concentration of the sample is determined from the standard curve and then heated to denature the proteins of the sample. Electrophoresis and membrane transfer are then performed. We use the first antibody to react with the PVDF membrane overnight, put down the second antibody the next day and finally rinse with PBS solution. The gels were then scanned using an automated gel imaging system and analysed for calculation using ImageJ software. Main antibodies and dilution ratio: Anti-Akt (ab81283, Abcam, 1:1000); Anti-beta Amyoid 1-42 (ab201060, Abcam, 1:1000); Anti-IL-1beta (ab9722, Abcam, 1:800); Anti-IL-10 (ab9969, Abcam, 1:800); Anti-PI3K (ab191606, Abcam, 1:1000); Anti-GFAP (BA0056, BOSTER, 1:800); Anti-IBA-1 (PB0517, BOSTER, 1:800); Anti-Beclin1 (ab207612, Abcam, 1:1000); Anti-LC3 (ab192890, Abcam, 1:1000); Anti-p-Akt (ab8805, Abcam, 1:1000); Anti-β-actin (GB12001, Servicebio, 1:2000).

### 2.10 Statistics

The data was processed by GraphPad Prism8.0 statistical software and the experimental results were analyzed. The measurement data were expressed as mean ± standard deviation (Mean ± SD). The comparison of the escape latency of mice in each group of behavior was performed by two-way ANOVA, and the comparison between the other groups was performed by One-way analysis of variance (one-way ANOVA). Taking α = 0.05 as the test level, and *p* ≤ 0.05 as statistically significant.

## 3 Results

### 3.1 Screening and target prediction results of active ingredients of LWD

**FIGURE 1:**
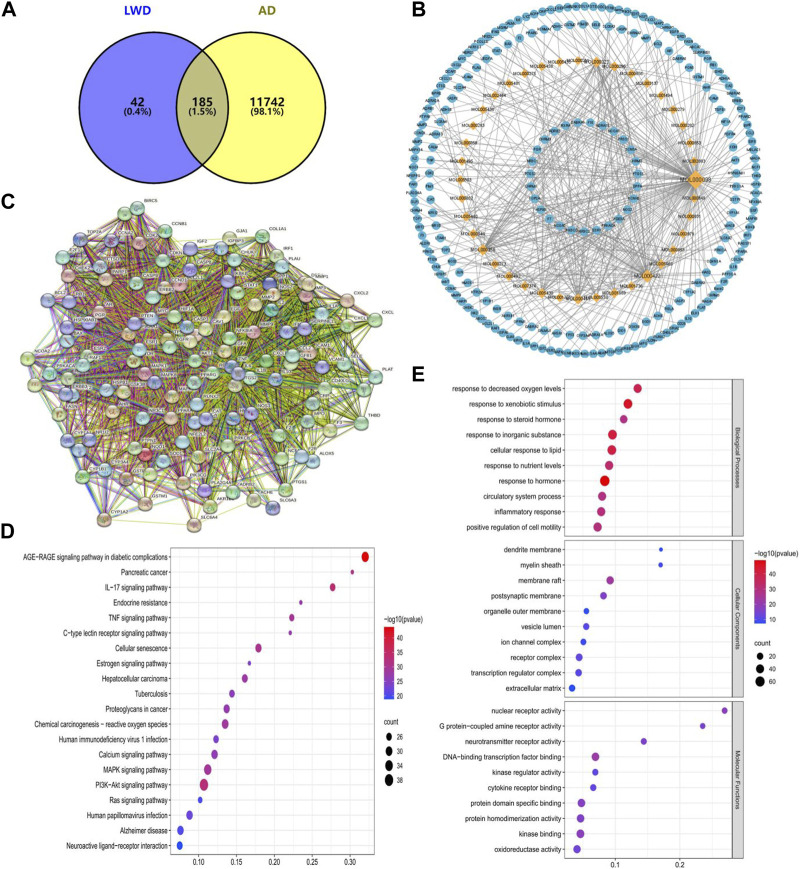
Network pharmacological prediction of LWD therapeutic AD targets. **(A)** Venny diagram of the common target of LWD and AD disease. **(B)** Compound-disease-target networks of LWD against AD. The diamond-shaped nodes represent the components of LWD, and the circular nodes represent the targets of LWD. **(C)** Core protein-protein interactions are identified by STRING software. **(D)** KEGG pathway enrichment analysis. **(E)** GO enrichment analysis of intersecting target biological processes, cellular components and molecular functions.

In this study, 40 compounds were obtained from the TCMSP database that fulfilled the conditions in the LWD, corresponding to 227 predicted targets. Screening against the OMIM database and GeneCards database finally revealed 185 relevant targets in the LWD that are associated with the treatment of AD (Figure 1A). To further interpret the target prediction results, we constructed a compound-target network containing 267 nodes (40 candidate compounds and 185 relevant targets). The network shows that most compounds have multi-target effects and exhibit multiple therapeutic effects (Figure 1B). We further filter meaningful targets based on the “drgee” value, and construct a PPI containing 130 nodes and 3112 edges based on a string database, with an average nodality degree of 47.9, an average local clustering coefficient: 0.686, and a PPI enrichment *p*-value<1.0E-16, indicating that proteins as a population are at least partially biologically connected and have a high degree of enrichment and interaction (Figure 1C). At the same time, in the enrichment analysis of KEGG and GO pathways, we performed the enrichment analysis of the KEGG pathway to test the possible targets of LWD in the treatment of AD. The first 20 pathways were identified, many of which were closely related to AD, and the relevant targets in LWD were mainly concentrated in PI3K-Akt pathway, cellular aging pathway, inflammatory pathway, MAPK pathway and Alzheimer’s disease-related pathway, among which PI3K-related pathway enriched with relevant targets as many as 38 (Figure 1D). The results of GO enrichment analysis were analyzed from three aspects: biological process, molecular function, and cell composition, and the targets related to AD in biological processes were mainly enriched in oxidative reaction, inflammation and circulatory system, targets related to AD in cell components were mainly enriched in synaptic parts, transcription factor parts and organelle outer membrane parts, and targets related to AD in molecular functions were mainly enriched in G protein-coupled receptors, nuclear receptor activity, neurotransmitter receptor activity and oxidoreductase activity (Figure 1E).

### 3.2 LWD can improve the learning and memory abilities of APP/PS1 mice

**FIGURE 2:**
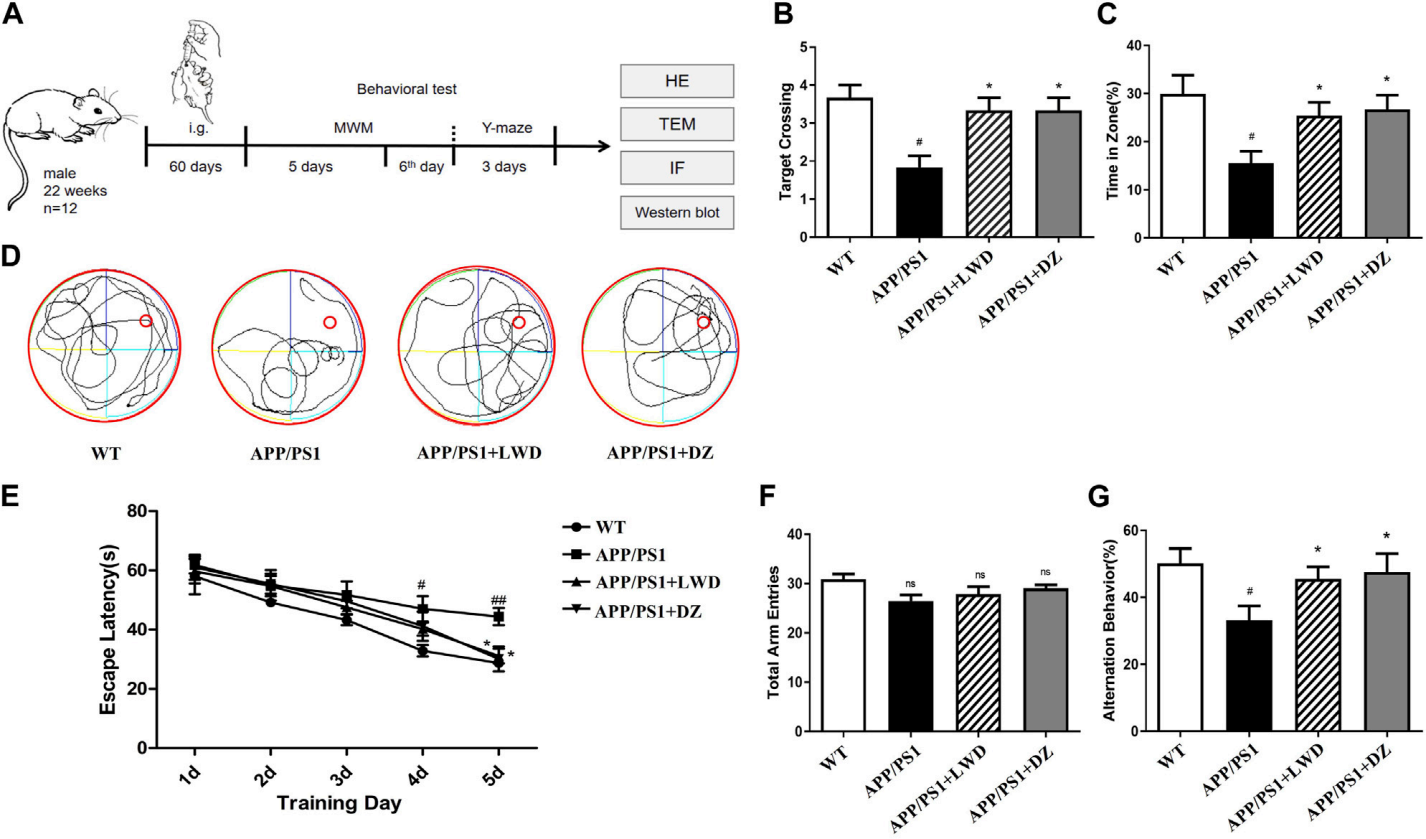
LWD improves long-term and short-term spatial learning and memory deficits in APP/PS1 mice in behavioral tests **(A)** Drug treatment time and basic experimental methods. **(B)** Average escape latencies of mice in acquisition trial phase from training day 1 to day 5. **(C,D)** Percentage of swimming time in target quadrant and frequency of platform crossing (swimming tracks of mice) in the probe trial phase on the sixth day. **(E)** One-minute swimming trajectory of mice in the MWM test on the sixth day. **(F,G)** In the Y maze test, the total number of arm advances and the percentage of spontaneous accurate alternation rate. (*n* = 12, compared with the WT group, ^*#*^ *p* < 0.05, ^*##*^ *p* < 0.01; compared with the APP/PS1 group, ^*^ *p* < 0.05, ^**^ *p* < 0.01).

In the Morris water maze, compared with the WT group, the escape latency of the APP/PS1 group was prolonged on the 4^th^ and 5^th^ day (*p* < 0.01), and it was significantly shortened after the intervention of LWD. Compared with the WT group, the APP/PS1 group had significantly fewer platform crossings and the percentage of stay time in the target quadrant. After the intervention of LWD, the number of crossing platforms and the percentage of stay time in the target quadrant increased (Figures 2B–E). Moreover, the results of the Y maze experiment showed that there was no significant difference in the total number of arm access times of each group. There were differences in the spontaneous alternation accuracy rate of mice in each group. Compared with the WT group, the spontaneous alternation accuracy rate of the APP/PS1 group is lower (*p* < 0.05); after LWD intervention, the spontaneous alternation accuracy rate has increased, and the difference is statistically significant (*p* < 0.05) (Figures 2F, G).

### 3.3 LWD improves the neuronal morphology in the hippocampus of APP/PS1 mice

The results of HE staining showed that the hippocampal nerve cells in the WT group had complete structure, neatly arranged neuronal cells, and clear nuclear staining, while the APP/PS1 group had structural abnormalities in the hippocampus, with significantly reduced number of nerve cells, loose arrangement, and disordered cell bands. At the same time, there were many neuropathological changes such as nucleus pyknosis and deepening of staining under the visible light microscope; after the intervention of LWD and DZ, the histopathological results of hippocampus were improved. The cells show a more regular arrangement and more uniform distribution, as well as clearer and normal structure.

**FIGURE 3:**
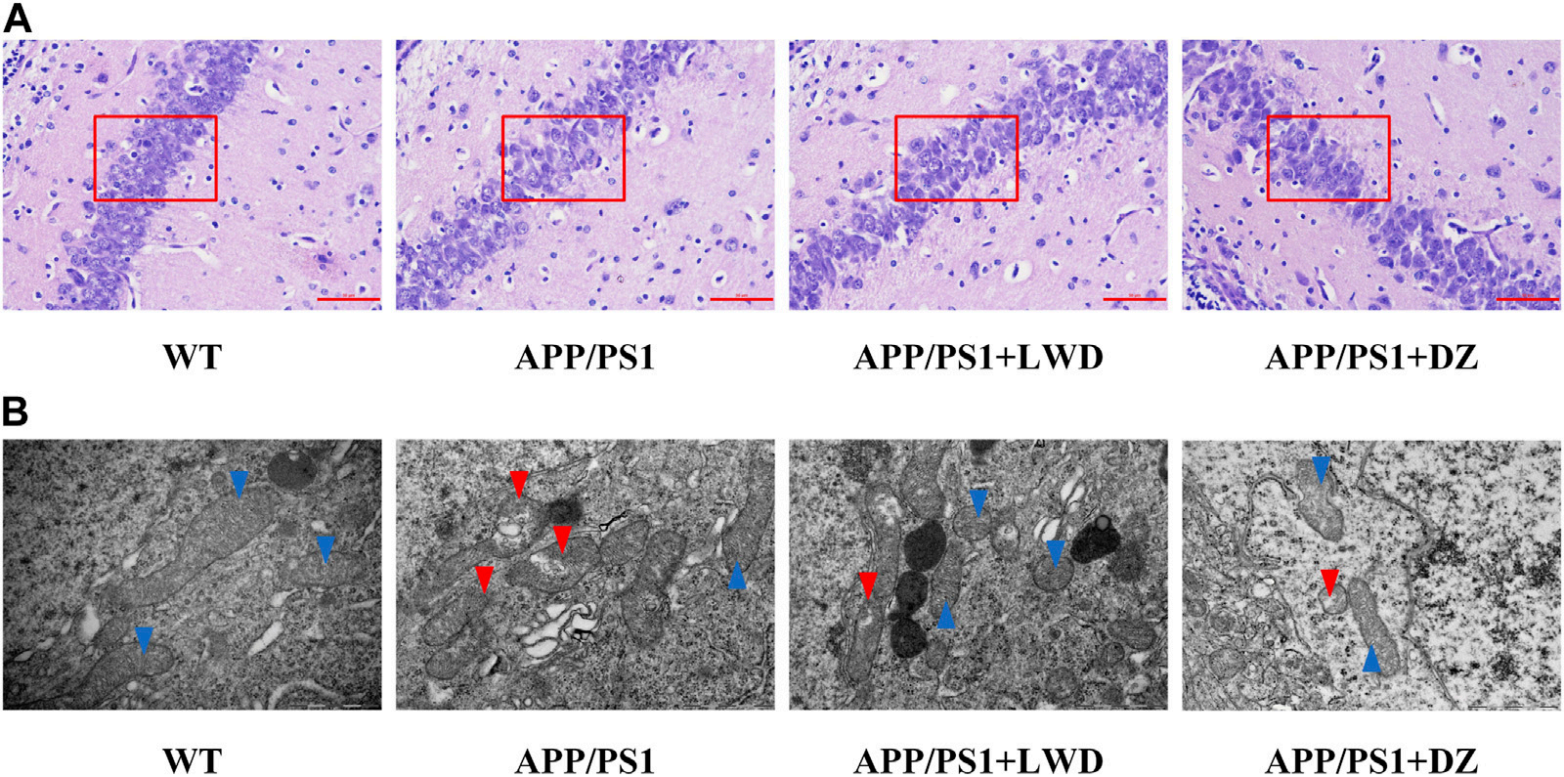
LWD improves neuronal morphology in APP/PS1 mice. **(A)** Neuronal cell structure in the hippocampus of each group of mice. **(B)** Neuron ultrastructure in the hippocampus of each group of mice. Blue arrows rIGUREresent undamaged mitochondria and red arrows represent damaged mitochondria.

Mitochondria are the subcellular organelles of neurons. Accumulation of Aβ will destroy the structure of mitochondria and damage neurons. Transmission electron microscopy observation of the mitochondrial structure of each group of mice showed that the mitochondrial double-layered membrane structure of neurons in the WT group was intact, the mitochondrial cristae were clear, and the nuclear membrane structure was intact. The mitochondria with abnormal neuron structure in APP/PS1 group increased significantly, which manifested itself by the dissolution of the double-layer membrane structure, the destruction of the inner cristae, and a large number of degenerated mitochondria seen in the cells. The matrix became deep and accumulated to form myeloid bodies. After the intervention of LWD and DZ, most of the mitochondria were intact, some mitochondria were slightly swollen, and the nuclear membrane structure was relatively complete (Figures 3A, B).

### 3.4 LWD reduces the Aβ and inflammatory response in the hippocampus of APP/PS1 mice

**FIGURE 4:**
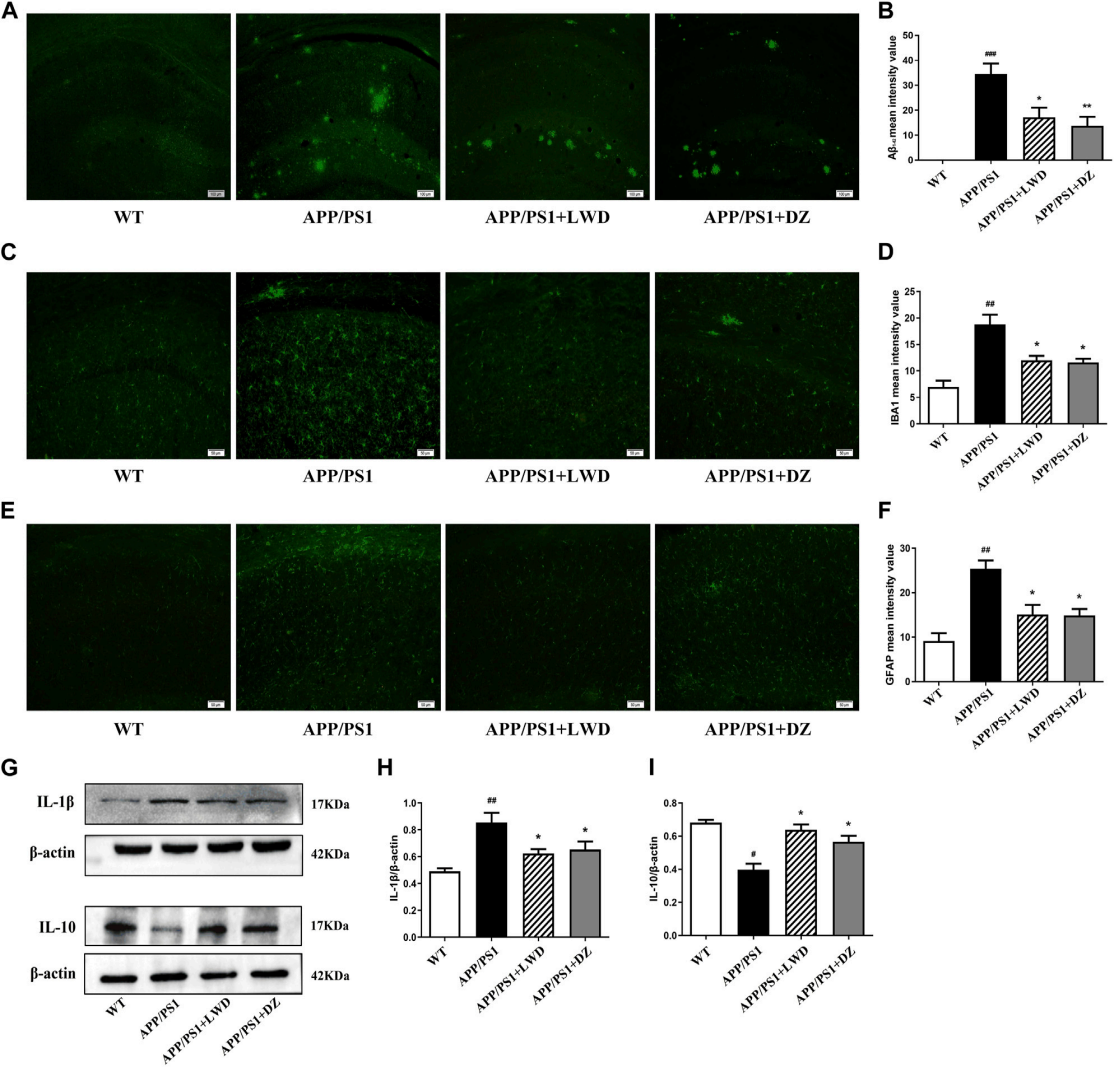
LWD reduced the Aβ and inflammatory response in the hippocampus of the APP/PS1 mice and the activated number of glial cells. **(A)** Positive expression of Aβ in each group in immunofluorescence. **(B)** Quantitative histogram of Aβ positive expression in each group. **(C)** Positive expression of IBA1 in each group in immunofluorescence. **(D)** Quantitative histogram of IBA1 positive expression in each group. **(E)** Positive expression of GFAP in each group in immunofluorescence. **(F)** Quantitative histogram of GFAP positive expression in each group. **(G–I)** Protein expression and quantitative histogram of pro-inflammatory factors IL-1β and IL-10 in each group. (*n* = 3, compared with the WT group, ^*#*^ *p* < 0.05, ^*##*^ *p* < 0.01, ^*###*^ *p* < 0.001; compared with the APP/PS1 group, ^*^ *p* < 0.05, ^**^ *p* < 0.01).

We found that compared with the WT group, the expression of Aβ1-42 in APP/PS1 group was significantly increased (*p* < 0.001); compared with the APP/PS1 group, the expression of Aβ1-42 in the LWD group and the DZ group was significantly decreased (*p* < 0.05). LWD can reduce the deposition of Aβ1-42 (Figures 4A, B).

GFAP is a unique skeleton protein of astrocytes and a sign of its activation. The activation of astrocytes can be assessed by detecting its positive expression. The results showed that, compared with the WT group, the positive expression of GFAP in the APP/PS1 group was increased. Compared with the APP/PS1 group, the GFAP expression in the LWD group and the DZ group was decreased (*p* < 0.05) (Figures 4C, D). As a marker of microglia activation, IBA1 was used to measure the activation of microglia. The results show that compared with the WT group, the positive expression rate of IBA1 in the APP/PS1 group was increased, indicating that the microglia were activated in a large amount. In the LWD group and the DZ group, the expression of IBA1 was decreased (*p* < 0.05) (Figures 4E, F).

The results further showed that the drug effect reduced the level of pro-inflammatory cytokines, with increased IL-1β expression and decreased IL-10 expression in APP/PS1 group. After treatment, the expression of IL-1β decreased and the expression of IL-10 increased in LWD group and the DZ group (*p* < 0.05). Therefore, we believe that LWD reduces inflammation in the hippocampus of mice by regulating the expression of inflammation-related factors (Figures 4G–I).

### 3.5 LWD improves autophagy in APP/PS1 mice

**FIGURE 5:**
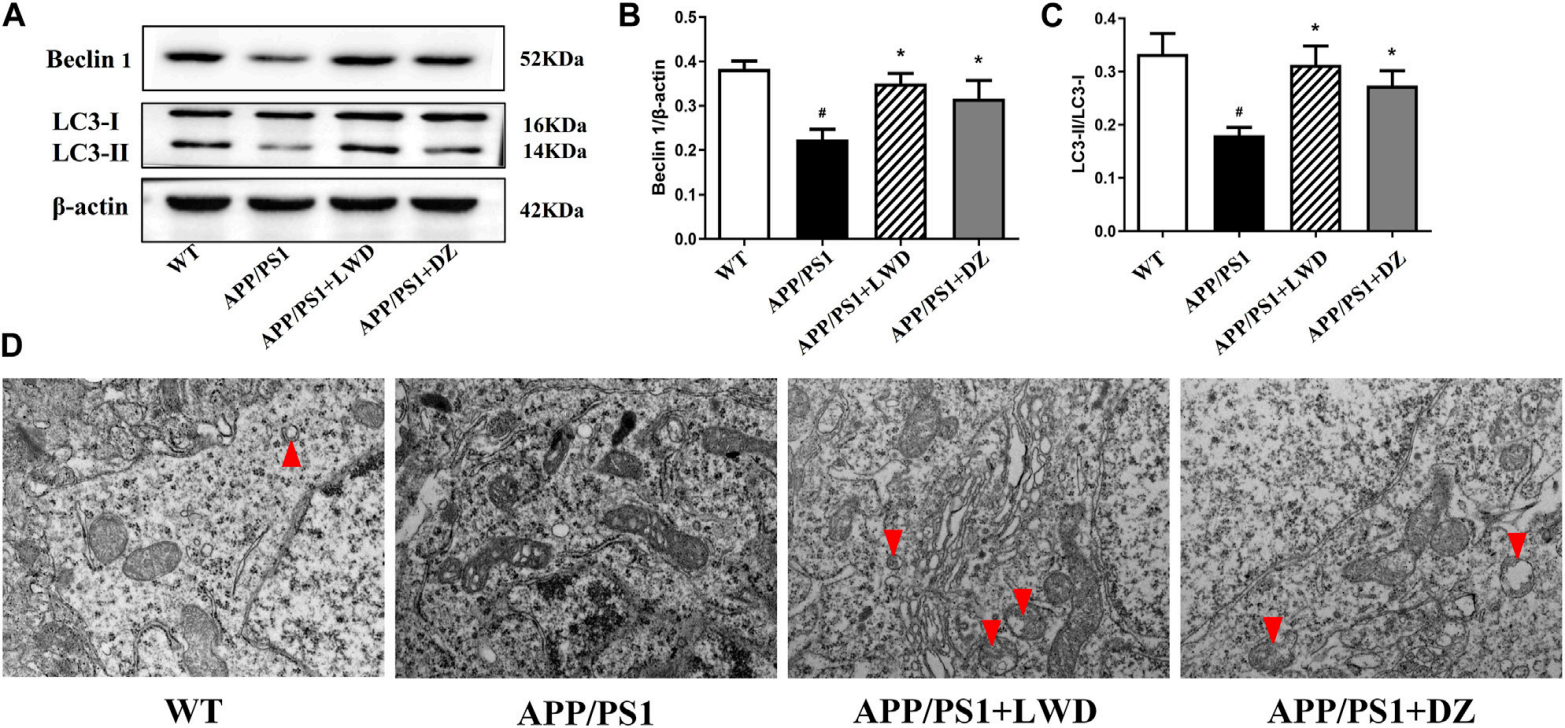
LWD increases autophagy activity in neurons in the brains of APP/PS1 mice. **(A–C)** Sample Western blots show the protein levels of LC3and Beclin1, actin as a loading control for each group (*n* = 3, compared with the WT group, ^#^ *p* < 0.05; compared with the APP/PS1 group, *p < 0.05). **(D)** TEM micrograph examples of autophagosomes in the hippocampal CA1 area. Red arrows indicate autophagy states and autophagosome. Scale bar, 500 nm.

Beclin 1 is a key regulator of autophagosome membrane formation. Compared with the WT group, the expression of Beclin1 in the APP/PS1 group was reduced (*p* < 0.05). After LWD and DZ intervention, compared with the APP/PS1 group, the expression of Beclin 1 has increased in the LWD and the DZ group (*p* < 0.05). Both LC3-I and LC3-II present in autophagy are considered to be molecular markers of autophagy in cells, the magnitude of the LC3-II/I ratio can estimate the level of autophagy. Compared with the WT group, the expression of the ratio of LC3-II to LC3-I in the APP/PS1 group is decreased, the difference is statistically significant (*p* < 0.05); after intervention, the expression of the ratio of LC3-II to LC3-I in the LWD group and the DZ group has increased (*p* < 0.05) (Figures 5A–C). The TEM results show that the number of autophagosome in the APP/PS1 group has almost zero. After the intervention of LWD and DZ, the number of autophagosome increased. (Figure 5D).

### 3.6 LWD regulates PI3K/Akt signaling pathway in APP/PS1 mice

**FIGURE 6:**
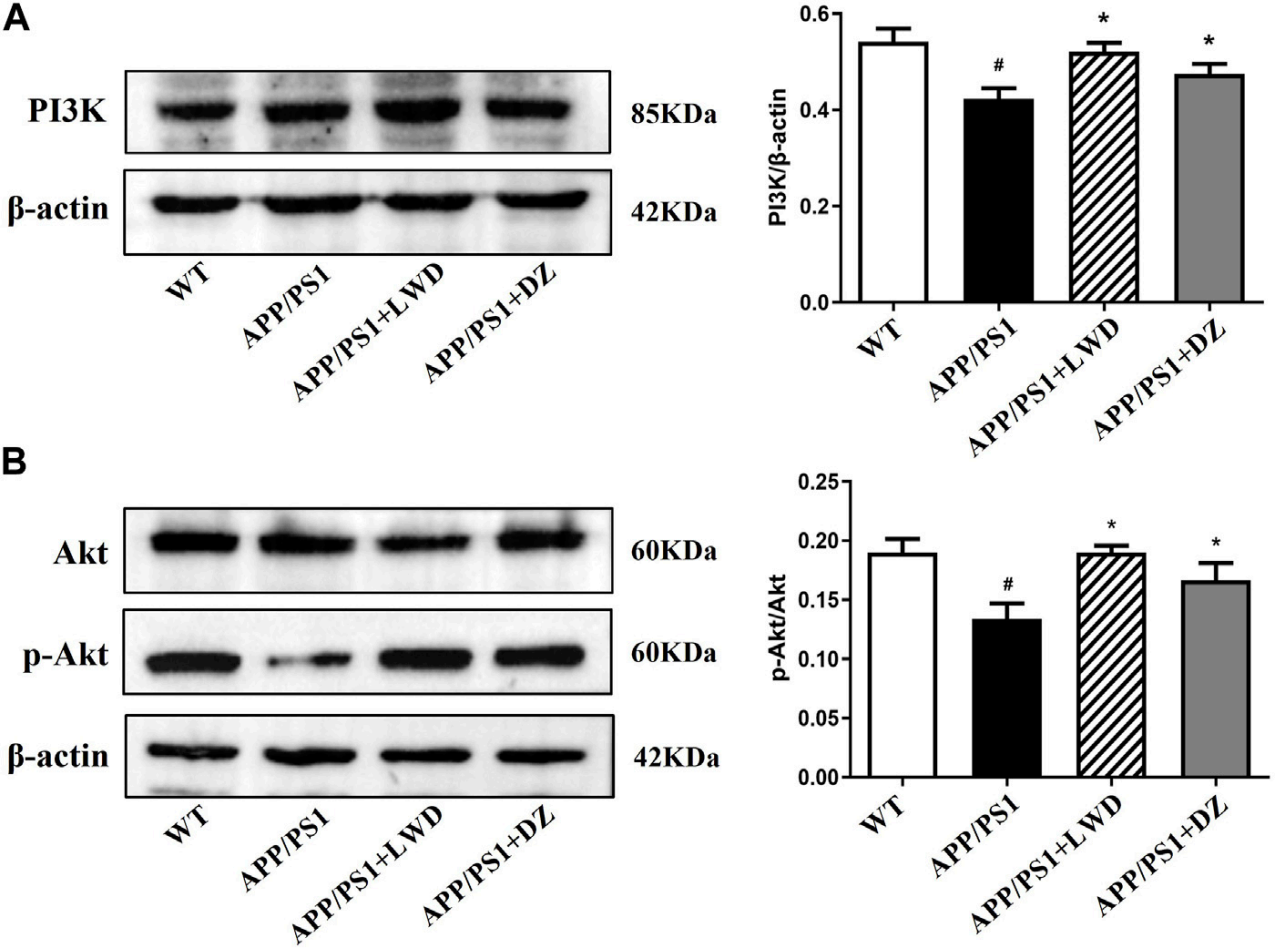
LWD changes the positive expression of PI3K/Akt signaling pathway related proteins in APP/PS1 mice. **(A)** Positive expression of PI3K protein in each group and quantitative histogram. **(B)** Positive expression and quantitative histogram of Akt and p-Akt protein in each group. (*n* = 3, Compared with the WT group, ^#^ *p* < 0.05; compared with the APP/PS1 group, *p < 0.05).

The PI3K-Akt signalling pathway is an intracellular signal transduction pathway that promotes cell survival, growth. This process is mediated by serine or threonine phosphorylation of a range of downstream substrates, of which the key genes being phosphatidylinositol 3-kinase (PI3K) and Akt. Compared with the WT group, the expression of PI3K in the APP/PS1 group was reduced (*p* < 0.05). Compared with the APP/PS1 group, the expression of PI3K increased in the LWD and DZ groups after the intervention (*p* < 0.05). Compared with the WT group, the expression of p-Akt/Akt in the APP/PS1 group was decreased (*p* < 0.05). Compared with the APP/PS1 group, the expression of p-Akt/Akt increased in the LWD and DZ groups after the intervention (*p* < 0.05) (Figures 6A, B).

## 4 Discussion

Traditional Chinese medicine believes that brain is the sea of medulla oblongata, the mansion of the primordial spirit, and people’s mental faculties and memory are dependent on brain. While the kidney is the foundation of innate nature and responsible for storing essence. The kidney is responsible for the production of bone and marrow, which is the innate foundation and root of life activities, and is closely related to the growth, development and aging of the human body. Kidney energy leads to the brain. Elderly person’s kidney qi is declining, the brain marrow is not nourished and the brain marrow is empty, and make memory loss, slow reaction, and further development for AD. Thus, the etiology of AD in the elderly lies in lack of essence in kidney.

In recent years, in the clinical treatment of dementia, traditional Chinese medicine “kidney governs the bones to produce marrow, and brain is the sea of marrow”. The theory of kidney therapy has gradually been recognized. Deficiency of the kidney essence results in slow blood flow, stagnation of qi and blood stasis, and then the brain Insufficiency of qi and blood causes the symptoms of dementia and mental decline (Wang et al., 2016a; Wang et al., 2016b; Zhang et al., 2017). LWD nourishes the kidney, liver and spleen, and mainly supplements kidney yin and essence. Experimental studies have shown that LWD can scavenge free radicals, reduce lipid peroxide levels, increase the activity of superoxide dismutase, regulate the level of sex hormones in the body, and nourish yin and nourish the kidney. It is an important mechanism for effectiveness of anti-ageing treatment (Bai et al., 2022).

In this study, we first observed whether LWD can improve the learning and memory abilities and working memory of the animals after the intervention of the APP/PS1 mice by Morris water maze experiment and Y maze experiment, and explored whether it can improve cognitive function. The results showed that APP/PS1 mice showed obvious learning and working memory impairments in both Morris water maze and Y maze experiments. After the intervention of LWD, the learning and memory abilities and working memory abilities were improved, and cognitive dysfunction has improved.

In network pharmacology analysis, 40 LWD-related compounds and 185 AD-related targets were screened by network pharmacology, and after PPI network analysis, the relationship between related targets was highly enriched and highly interacting. In KEGG and GO enrichment analysis, we found that the relevant targets in LWD were mainly concentrated in PI3K-Akt pathway, cellular aging pathway, inflammation pathway, MAPK pathway and AD-related pathway, which PI3K-related pathway was enriched with relevant targets. In GO enrichment analysis, we analyze biological processes, cell components, molecular functions, and AD-related targets mainly focus on oxidative reactions, inflammation, neuronal correlation, synaptic parts and a series of parts, and we believe that there is a certain correlation between the results of KEGG pathway enrichment analysis and GO enrichment analysis, and these are very important parts for AD.

The stability of Aβ levels in brain depends on the balance between Aβ production and clearance. Studies have shown that excessive accumulation of Aβ can induce oxidative stress, inflammation, and the degeneration of brain-derived neurotrophic factors, further aggravating the pathological process (Smith et al., 2000; Lacor et al., 2007). In this part of the experiment, the positive expression of Aβ1-42 in the hippocampus of mice was detected by immunofluorescence chemical method and the average optical density was analyzed to explore whether LWD could reduce the deposition of Aβ1-42 in the brain. The experimental results showed that compared with the WT group, the expression of Aβ1-42 in APP/PS1 group was significantly increased as expected. However, after the treatment with LWD, Aβ expression decreased, which proved that the intervention of LWD could reduce the expression level of Aβ1-42 in the brains of APP/PS1 transgenic mice.

Glial cells are the most important immune defense line of the central nervous system (Qi and Wang, 2020). In the pathological condition of AD, the clearance of Aβ is largely dependent on the phagocytosis of glial cells in the brain. However, in the occurrence of inflammation stage of AD, the physiological function of glial cells is impaired (Misra et al., 2020; Wu et al., 2020). Although Aβ can bind to the receptor protein on the surface of glial cells, enter the cell through endocytosis, and be cleared by microglia, but it also causes Glial cells that are in an abnormally activated state to release neuroinflammatory factors and inflammatory mediators, further aggravating the local inflammatory response, and causing irreversible damage to neurons. Glial fibrillary acidic protein (GFAP), as a unique skeleton protein of astrocytes, is a marker of their activation, and Calcium Binding Adapter Molecule1 (IBA1) is a marker protein of microglia activation (Zhang, L. et al., 2019). In the results of this experiment, the fluorescence expression of IBA1 and GFAP was enhanced after treatment with LWD, indicating that microglia and astrocytes are less activated after treatment. Meanwhile, the expression of pro-inflammatory factors decreased and the expression of anti-inflammatory factors increased after LWD treatment, suggesting that LWD can play a role in improving ad by clearing performance and inhibiting inflammation.

Autophagy plays an important biological function in organisms. Misfolded proteins and ruptured cell debris that appear in the process of material metabolism in the body can be cleared through the autophagy pathway (Kroemer, 2015). With the deepening of research on autophagy and AD, it has been found that autophagy is one of the main ways to degrade and remove abnormally deposited proteins in the body. Autophagy is also closely related to the removal of Aβ (Mcquail et al., 2015). In AD patients, the autophagy function is significantly inhibited, resulting in a decrease in its ability to clear abnormal proteins. Studies have confirmed that the regulation of brain autophagy in AD patients is similar to that of animal models (D’Assante et al., 2017; Chen et al., 2014). Beclin1 and LC3 are the main proteins related to autophagy in mammalian cells. Beclin1 is the earliest key regulator in the study of autophagy. It binds to ligands and regulates the formation of autophagosome membranes and regulates intracellular autophagy activity (Asgari et al., 2020). LC3 is located in the inner membrane of autophagic vesicles, and its expression product LC3 is activated along with the process of autophagy, and combines with phosphatidylethanolamine to form membrane type LC3-II. The quantity is considered to be a specific and sensitive marker molecule for judging autophagy activity (Salminen et al., 2013). The results showed that LWD could significantly increase the expression of autophagy-related proteins Beclin1 and LC3-Ⅱ/LC3-Ⅰ in APP/PS1 mice, and induce autophagy. The standard for detecting autophagosomes is electron microscopy (Xu et al., 2020). In this study, the expression level of neuronal autophagosomes in the hippocampus of each group of mice was detected by transmission electron microscopy at the same time. Under the electron microscope, the results showed that compared with the WT group, multiple autophagosomes can be seen in the visual field after the intervention of LWD, which is consistent with the Western Blot detection of autophagy-related proteins Beclin1 and LC3-Ⅱ/LC3-Ⅰ.

According to our network pharmacology experiments, PI3K/Akt pathway is closely related to AD. Abnormal PI3K/Akt signaling pathway have been proven to be associated with pathological changes of a variety of human neurodegenerative diseases (Dhadve et al., 2020). The PI3K/Akt signaling pathway is activated by a variety of biological factors that regulate the activation of downstream-associated proteins and exert important biological effects, including the regulation of neuroinflammation and autophagy. PI3K is a heterodimer of the p110 catalytic subunit Heterodimer of a complex formed with the p85 regulatory subunit, and an important signal transducer for cell metabolism, proliferation and apoptosis. It plays a key role in enabling AktThr308 site phosphorylation activation-dependent pathways. This pathway was demonstrated in AD to regulate downstream proteins to ameliorate neuroinflammation, oxidative stress and other influences on disease development (Ma et al., 2014; Cai et al., 2018; Gleason et al., 2019). Since the PI3K/AKT signalling pathway is neural protective, activation of this pathway facilitates the treatment of neurodegenerative diseases such as AD, which is the opposite of its manifestation in cancer. Studies have shown that regulating inflammation and apoptosis signals by regulating the PI3K/Akt signaling pathway can improve the learning and memory ability of C57/BL mice (Bossler et al., 2019). However, there are relatively few studies on the role of autophagy in the pathogenesis and treatment of AD through the PI3K/Akt signaling pathway.

Based on the PI3K/Akt signaling pathway to regulate inflammation and autophagy and its important role in the occurrence and development of AD (Zhou et al., 2016). Autophagy can also weaken the inflammatory response by promoting the clearance of apoptotic cells. Recent studies have found that during the apoptosis stage, autophagy is also activated (Saitoh et al., 2008). If these cells are missing or inhibited from autophagy, the clearance of apoptotic cells will be insufficient, which will lead to secondary necrosis of dead cells (Nakahira et al., 2015). Therefore, the activation of autophagy reduces the inflammatory response by preventing the occurrence of secondary necrosis. However, the possible mechanism of the interaction between autophagy and inflammatory response still needs to be further explored, and this part will be carried out in follow-up experiments. The results showed that compared with APP/PS1 group, the expression levels of PI3K and p-Akt increased after the intervention of LWD, which proved the molecular biological mechanism of the neuroprotective effect of LWD, which may be one of its mechanisms of preventing AD.

In conclusion, this study demonstrated that LWD effectively improved the learning and memory ability of AD mice, and the mechanism of LWD may be related to the activation of the autophagy pathway regulated by the PI3K/Akt signaling pathway, the attenuation of glial cell activation-induced inflammatory response and the upregulation of the expression of Beclin1 and LC3B. The results of this study provide a scientific theoretical basis for further deepening the clinical application of LWD in the prevention and treatment of AD, but its detailed mechanism of action still needs to be further explored.

## Data Availability

The original contributions presented in the study are included in the article/supplementary material, further inquiries can be directed to the corresponding authors.
